# A cross-sectional study on the association of anxiety and depression with the disease activity of systemic lupus erythematosus

**DOI:** 10.1186/s12888-022-04236-z

**Published:** 2022-09-05

**Authors:** Jiafen Liao, Jin Kang, Fen Li, Qi Li, Jia Wang, Qi Tang, Ni Mao, Shu Li, Xi Xie

**Affiliations:** 1 Department of Rheumatology, the Second Xiangya Hospital of Central South University, Changsha, 410011 Hunan China; 2 Department of Cardiology, Heart Center of Henan Provincial People’s Hospital, Henan Key Laboratory for Coronary Heart Disease Prevention and Control, Central China Fuwai Hospital of Zhengzhou University, Zhengzhou, Henan China

**Keywords:** Systemic lupus erythematosus, Depression, Anxiety, Disease activity, SLEDAI

## Abstract

**Background:**

Systemic Lupus Erythematosus (SLE) is an autoimmune disease that affects multiple systems and increases the risk of mental disorders such as depression and anxiety. We conducted an observational, single-center, cross-sectional study to investigate the relationship between depression, anxiety, and SLE disease activity.

**Methods:**

The Patient Health Questionnaire 9 (PHQ-9) was used to assess depression, and the 7-item Generalized Anxiety Disorders Scale was used to assess anxiety (GAD-7). Using the chi-square/exact Fisher's tests, socio-demographic data, clinical and other characteristics of SLE patients were compared between depression or anxiety and non-depression/non-anxiety groups. To identify optimal levels of Systemic Lupus Erythematosus Disease Activity Index (SLEDAI) for predicting depression or anxiety, receiver-operator curves (ROC) were drawn.

**Results:**

Among the 325 patients involved in this study, patients with depression or anxiety had significantly higher SLE activity (*p* < 0.001), and more frequent musculoskeletal (*p* < 0.05) and neuropsychiatric symptoms (*p* < 0.05). Depression and anxiety are more common in the moderate-severe active group than in the inactive-mild active group (depression: OR 3.350, 95%CI 2.015, 5.570, *p* < 0.001; anxiety: OR 4.085, 95%CI 2.493, 6.692, *p* < 0.001). The optimal SLEDAI cutoff value of 8.5 predicted depression with a sensitivity of 50.5% and a specificity of 78.4% (AUC 0.660, *p* < 0.001) and anxiety with a sensitivity of 54.2% and a specificity of 78.4% (AUC 0.684, *p* < 0.001).

**Conclusion:**

SLE disease activity is positively associated with the severity of depression and anxiety. Those patients whose SLEDAI scores are greater than 8.5 are more likely to suffer from mental disorders which require additional attention to them.

## Background

Systemic Lupus Erythematosus (SLE) is a chronic autoimmune disease that affects multiple organs and systems. However, apart from the somatic damage, there is an increased risk of mental disorders in SLE patients, including depression and anxiety. A number of studies have been carried out to evaluate the prevalence of depression and anxiety in SLE patients, and results show a wide range of prevalence rates ranging from 2.1–78.6% and 2.9–84.9%, respectively [1]. However, the diagnosis of depression and anxiety in SLE patients is usually delayed or missed in regular clinical practice.

What’s more, it is demonstrated by plenty of research that the interplay between depression, anxiety, and SLE can lead to an increased incidence of suicidal ideation, poor adherence to treatment, and increased functional disability [2, 3] . These factors ultimately play an important role in decreasing patients’ quality of life [4–6] . With the high prevalence and risk of these mental disorders in SLE patients, it is crucial to early identify patients with anxiety and depression.

There have been numerous researches being intended to figure out the impact factor of depression and anxiety in SLE patients. And a number of factors have been reported to contribute to the higher prevalence. Among them, disease activity is the most frequently explored. However, whether the relationship between depression, anxiety, and disease activity is definite or not is still controversial. Some studies have reported that greater disease activity is linked to a greater risk of depression and anxiety, while others found no association between higher SLE activity and the occurrence of these symptoms.

In light of this, we conducted an observational, single-center, cross-sectional, and descriptive study in patients with SLE, in order to find out the relationship between depression, anxiety, and disease activity.

## Methods

### Study population

This cross-sectional study was conducted between 10 – 17 May 2021 in the outpatient clinic and inpatient department of the second Xiangya Hospital, Central South University, China. Participants in this study were previously diagnosed with SLE according to the 2012 SLICC criteria. The inclusion criteria were (a) age 18 or older; (b) volunteer to participate in this survey; (c) capable of reading and writing; (d) capable of finishing the questionnaire survey by smartphone independently. We excluded participants if they had any of the following items: (a) history of depression or anxiety before the diagnosis of SLE; (b) history of the treatment of mental disease; (c) history of substance abuse; (d) serious disorders of heart, liver, kidney, or other major organs; (e) diagnosed with any disease or impairment that might prevent them from completing the questionnaire independently. All of the participants gave written informed consent and the study was approved by the Second Xiangya Hospital local ethics committee.

### Data collection

The questionnaire includes 3 sections. The opening section is socio-demographic data including age, gender, educational levels, annual household income, time of onset of SLE, and history of smoking and drinking. The second section is the SLE disease activity assessment, and the third section is the mental disease activity assessment. It took about 3–5 min for participants to complete the questionnaire.

#### SLE disease activity assessment

SLE disease activity was assessed by SLE Disease Activity Index 2000 (SLEDAI-2000) [7] . The SLEDAI-2000 tool is a cumulative and weighted index used to assess disease activity across 24 separate disease descriptors in patients with SLE. All SLE-related descriptors that are present at the time of the visit or within the previous 10 days should be checked off on the form. A total score can fall between 0 and 105, with a higher score representing a more significant degree of disease activity. The results were divided into 4 levels based on the scores: a score between 0–4 is considered disease inactive, a score between 5–9 is considered mildly active, a score between 10–14 is considered moderate activity, and a score of more than 15 is considered severe activity.

#### Assessment of anxiety/depression

Anxiety was measured by the 7-item Generalized Anxiety Disorders Scale (GAD-7). GAD-7 consists of seven items measuring anxiety symptoms. Each item is scored on a four-point Likert scale (0–3) with higher total scores reflecting greater anxiety severity. The following cut-offs correlate with the level of anxiety severity: a score between 0–4 is considered to be non-anxiety, a score between 5–9 is considered mild anxiety, a score between 10–14 is considered moderate anxiety, and a score more than 15 is considered severe anxiety. The GAD-7 has shown good reliability and construct validity [8] .

Depression was measured by the Patient Health Questionnaire 9 (PHQ-9). PHQ-9 consists of nine items measuring depressive symptoms corresponding to the diagnostic criteria for major depressive disorder. Each item is scored on a four-point Likert scale (0–3) with scores ranging from 0–27, with higher scores reflecting greater depression severity. PHQ-9 scores more than 0, 5, 10, and 15 represented non-depression, mild, moderate, and severe depression, respectively. PHQ-9 has shown good psychometric properties [9] .

### Statistical analysis

Categorical data were summarized as counts and percentages, while continuous data were reported as the mean and standard deviation. Categorical variables were compared with the chi-square/Fisher’s exact tests, while continuous variables were compared with the Student’s *t*-test. Receiver-operator curves (ROC) were drawn to identify optimal levels of SLEDAI for predicting depression or anxiety. The statistical analysis was performed using SPSS software for Windows Version 19.0 (SPSS Inc., IL, USA). A *p*-value less than 0.05 was considered statistically significant.

## Results

### Characteristics of study subjects

This study enrolled 325 patients. Among them, 61.5% of patients (200/325) had a score reflecting the level of depression with the use of cutoff points in PHQ-9, while 54.4% of patients (177/325) had a score reflecting the level of anxiety with the use of cutoff points in GAD-7. Table 1 summarized the demographic and clinical characteristics. Patients with depression or anxiety significantly more often had moderate-severe SLE activity (*p* < 0.001, *p* < 0.001, respectively), musculoskeletal (*p* = 0.002, *p* = 0.023, respectively) and neuropsychiatric symptoms (*p* = 0.002, *p* < 0.001, respectively). Patients with depression also more often had lower than 50,000 RMB in annual household income compared with those without depression(*p* = 0.005). Meanwhile, we found no difference in age, gender, educational levels, time of onset of SLE, history of smoking, and drinking between the depression or anxiety group and non-depression/non-anxiety group.

**Table 1:** Demographic and clinical characteristics of SLE patients

	**Depression(*N* = 200)**	**Non-depression(*N* = 125)**	**Total**	**χ**^**2**^***/ t***	***p* value**	**Anxiety(*N* = 177)**	**Non-anxiety(*N* = 148)**	**Total**	**χ**^**2**^***/ t***	***p* value**
**Age**	44.57 ± 11.78	44.42 ± 12.12	44.51 ± 11.89	0.113	0.910	43.87 ± 11.92	45.28 ± 11.86	44.51 ± 11.89	-1.062	0.289
**Gender**				0.263	0.608				0.042	0.873
Male	13(0.07)	10(0.08)	23(0.07)			13(0.07)	10(0.07)	23(0.07)		
Female	187(0.94)	115(0.92)	302(0.93)			164(0.93)	138(0.93)	138(0.42)		
**Educational levels**				0.141	0.707				0.689	0.407
High school degree or less	121(0.61)	73(0.58)	194(0.60)			102(0.58)	92(0.62)	92(0.28)		
Bachelor's degree or above	79(0.40)	52(0.42)	131(0.40)			75(0.42)	56(0.38)	56(0.17)		
**Annual household income**				8.020	0.005				2.170	0.414
Less than 50,000	147(0.74)	73(0.58)	220(0.68)			126(0.71)	94(0.64)	94(0.29)		
More than 50,000	53(0.27)	52(0.42)	105(0.32)			51(0.29)	54(0.36)	54(0.17)		
**Disease activity**				22.790	0.000				32.996	0.000
Inactive-mild active	104(0.52)	98(0.78)	202(0.62)			85(0.48)	117(0.79)	202(0.62)		
Moderate-severe active	96(0.48)	27(0.22)	123(0.38)			92(0.52)	31(0.21)	123(0.38)		
SLEDAI 2000 score	13.61 ± 14.93	6.78 ± 5.16	10.98 ± 12.58	4.924	0.000	14.68 ± 15.63	6.56 ± 4.46	10.98 ± 12.58	6.110	0.000
**Anti-dsDNA antibody**										
Positive	20 (0.10)	7 (0.06)	27 (0.08)	1.955	0.215	19 (0.11)	8 (0.05)	27 (0.08)	3.005	0.106
Negative	180 (0.9)	118 (0.94)	298 (0.92)			158 (0.89)	140 (0.95)	298 (0.92)		
**Time of onset**				0.250	0.875				0.381	0.571
Within 5 years	99(0.50)	63(0.50)	162(0.50)			91(0.51)	71(0.48)	162(0.5)		
More than 5 years	101(0.51)	62(0.50)	163(0.50)			86(0.49)	77(0.52)	77(0.24)		
**History of smoking**				0.062	0.438				3.436	0.064
Yes	8(0.04)	3(0.02)	11(0.03)			9(0.05)	2(0.01)	2(0.01)		
No	192(0.96)	122(0.98)	314(0.97)			168(0.95)	146(0.99)	146(0.45)		
**History of drinking**				0.628	0.428				1.359	0.238
Yes	6(0.03)	2(0.02)	8(0.02)			6(0.03)	2(0.01)	2(0.01)		
No	194(0.97)	123(0.98)	317(0.98)			171(0.97)	146(0.99)	146(0.45)		
Clinical manifestation*				3.511	0.898				4.299	0.829
Skin and mucosa^a^	54(0.27)	29(0.23)	83(0.26)	0.584	0.445	46(0.26)	37(0.25)	83(0.26)	0.041	0.839
Musculoskeletal^b^	58(0.29)	18(0.14)	76(0.23)	9.152	0.002	50(0.28)	26(0.18)	76(0.23)	5.133	0.023
Hematological^c^	33(0.17)	18(0.14)	51(0.16)	0.256	0.613	34(0.19)	17(0.11)	51(0.16)	3.633	0.057
Lupus nephritis^d^	43(0.22)	17(0.14)	60(0.18)	3.189	0.074	39(0.22)	21(0.14)	60(0.18)	3.295	0.069
Neuropsychiatric^e^	87(0.44)	33(0.26)	120(0.37)	9.658	0.002	83(0.47)	37(0.25)	120(0.37)	16.587	0.000

^*^ Clinical manifestation included in this table was based on SLEDAI 2000 score

^a^Skin and mucosa: rash, alopecia, mucosal ulcers

^b^Musculoskeletal: arthritis and myositis

^c^Hematological: thrombocytopenia, leukopenia

^d^Lupus nephritis: Urinary casts, hematuria, proteinuria, and pyuria

^e^Neuropsychiatric: seizure, psychosis, organic brain syndrome, visual disturbance, cranial nerve disorder, lupus headache, CVA

### The relationship between SLE disease activity and depression or anxiety

**Fig. 1:**
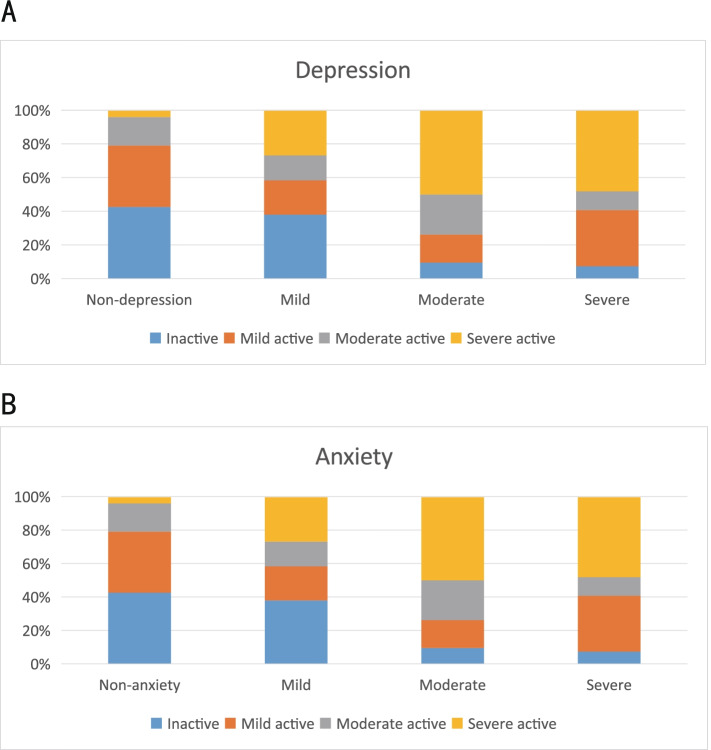
The relationship between SLEDAI and depression or anxiety. 1A. The relationship between SLEDAI and depression; 1B. The relationship between SLEDAI and anxiety

Among the patients involved in this study, the higher the disease activity, the higher the proportion of depression(Table 2, Fig. 1A) or anxiety(Table 3, Fig. 1B) in SLE patients, and the difference was statistically significant (*p* < 0.001).

**Table 2:** The relationship between SLE disease activity and depression in SLE patients

Disease activity	Non-depression (*N* = 125)	Depression			Total (*n* = 325)	χ^2^	*p*-value
		Mild (*n* = 103)	Moderate (*n* = 57)	Severe (*n* = 40)			
Inactive	55 (0.44)	38 (0.37)	12 (0.21)	5 (0.13)	110 (0.34)	60.54	0.001
Mild	43 (0.34)	30 (0.29)	12 (0.21)	7 (0.18)	92 (0.28)		
Moderate	18 (0.14)	17 (0.17)	14 (0.25)	5 (0.13)	54 (0.17)		
Severe	9 (0.07)	18 (0.17)	19 (0.33)	23 (0.58)	69 (0.21)

**Table 3:** The relationship between SLE disease activity and anxiety in SLE patients

Disease activity	Non-anxiety (*N* = 148)	Anxiety			Total (*n* = 325)	χ^2^	*p*-value
		Mild (*n* = 108)	Moderate (*n* = 42)	Severe (*n* = 27)			
Inactive	63 (0.43)	41 (0.38)	4 (0.1)	2 (0.07)	110 (0.34)	74.717	0.001
Mild	54 (0.36)	22 (0.2)	7 (0.17)	9 (0.33)	92 (0.28)		
Moderate	25 (0.17)	16 (0.15)	10 (0.24)	3 (0.11)	54 (0.17)		
Severe	6 (0.04)	29 (0.27)	21 (0.5)	13 (0.48)	69 (0.21)		

We further investigated the risk of depression or anxiety in SLE patients with different levels of disease activity (Table 4). Based on the SLEDAI score, we divided SLE patients into a moderate-severe active group and an inactive-mild active group. The risk of depression or anxiety in both groups is shown in Table 4. Prevalence of probable depression/anxiety based on PHQ-9/GAD-7 cut-off was significantly higher in the moderate-severe active group compared with those in the inactive-mild active group. (depression: OR 3.350, 95%CI 2.015, 5.570, *p* < 0.001; anxiety: OR 4.085, 95%CI 2.493, 6.692, *p* < 0.001).

**Table 4:** The risk of depression and anxiety in moderate-severe SLE compared with inactive-mild SLE

	Number of patients		Total	χ^2^	*p*-value	OR
SLE	Depression	Non-depression				
Moderate-severe active	96	27	123	22,79	0.00	3.350(2.015–5.570)
Inactive-mild active	104	98	202			
SLE	Anxiety	Non-anxiety				
Moderate-severe active	92	31	123	32.996	0.00	4.085(2.493–6.692)
Inactive-mild active	85	117	202			

### Predicted value of SLEDAI for depression or anxiety in patients with SLE

**Fig. 2:**
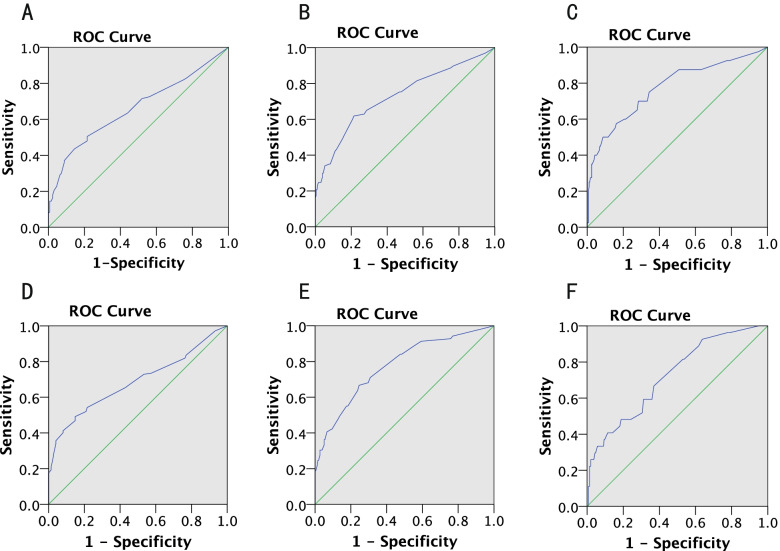
Receiver-operating characteristic (ROC) curve for SLEDAI in the diagnosis of depression or anxiety. ROC curve for SLEDAI in the diagnosis of mild/moderate/severe depression is shown in Fig. 2A, 2B, and 2C, respectively. ROC curves for SLEDAI in the diagnosis of mild/moderate/severe anxiety are shown in Fig. 2D, 2E, and 2F, respectively

ROC curve analysis demonstrated an AUC of 0.660 (*p* < 0.001) for SLEDAI as a predictor of depression (Table 5, Fig. 2). The SLEDAI score greater than 8.5 achieved 50.5% sensitivity and 78.4% specificity for predicting depression. Thus, when the SLEDAI score of SLE patients was less than 8.5, patients had a low risk of depression for the present. What’s more, when the SLEDAI score was higher than 8.5 and less than 10.5, patients were prone to mild depression (*p* < 0.001). When the SLEDAI score of SLE patients was higher than 10.5 and less than 14.5, patients were prone to moderate depression (*p* < 0.001). When the SLEDAI score of SLE was greater than 14.5, patients were prone to severe depression (*p* < 0.001).

**Table 5:** ROC analysis for SLEDAI in the diagnosis of depression and anxiety

	AUC	SE	*P* value	95% CI		Sensitivity	Specificity	Cut-off value of SLEDAI
				Lower limit	Upper limit			
Depression								
Non-mild	0.660	0.300	0.000	0.601	0.718	0.505	0.784	8.5
mild-moderate	0.729	0.033	0.000	0.664	0.793	0.619	0.785	10.5
moderate-severe	0.772	0.044	0.000	0.685	0.859	0.575	0.839	14.5
Anxiety								
Non-mild	0.684	0.029	0.000	0.627	0.742	0.542	0.784	8.5
mild-moderate	0.773	0.033	0.000	0.079	0.838	0.667	0.754	10.5
moderate-severe	0.722	0.050	0.000	0.624	0.821	0.481	0.774	14.5

As with ROC analysis for predicting anxiety, we found that the optimal SLEDAI cutoff value of 8.5 showed a sensitivity of 54.2% and a specificity of 78.4% (AUC 0.684, *p* < 0.001) (Table 5, Fig. 2). Thus, when the SLEDAI score of SLE patients was less than 8.5, patients had a low risk of anxiety in the present. What’s more, when the SLEDAI score was higher than 8.5 and lower than 10.5, patients were prone to mild anxiety (*p* < 0.001). When the SLEDAI score of SLE patients was higher than 10.5 and lower than 14.5, patients were prone to moderate anxiety (*p* < 0.001). When the SLEDAI score of SLE was higher than 14.5, patients are prone to severe anxiety (*p* < 0.001).

## Discussion

In the past years, depression and anxiety are rarely diagnosed early in their course in SLE, partially due to the lack of reliable and accepted screening metrics in this patient population, but also due to clinicians’ focus on the somatic symptoms. In a study by Mok et al., both depression and anxiety independently impacted the quality of life in SLE patients [10] . Therefore, finding out the prevalence of depression and anxiety in SLE patients allows us to make a better clinical decision with them. In this study, we analyzed depression and anxiety in SLE patients, and find that the prevalence of probable depression/anxiety based on PHQ-9/GAD-7 cut-off in SLE patients is 54.5% and 61.5%, respectively. These high ratios highlighted the importance of early diagnosis of these mental disorders in SLE patients.

In this study, we demonstrated that family income, disease activity, and manifestation in musculoskeletal and neuropsychiatric systems are related to depression. And in the meantime, disease activity, and manifestation in musculoskeletal and neuropsychiatric systems are associated with anxiety in patients with SLE. In previous studies, various factors have been linked to depression or anxiety in lupus, including age, antibody, fatigue, sleep quality, specific organ involvement, some cytokines, disease activity, glucocorticosteroid use, unemployment, and so on [11–17]. These diverse findings indicate that depression and anxiety in lupus are likely mediated through a complex mixture of biological, social, economical, psychological, and environmental contributors.

Of the numerous disease-related factors, the association between SLE disease activity and depression or anxiety remains one of the most frequently studied, though the results between studies are inconsistent. We demonstrated that the ratio of depression and anxiety varies in patients with different levels of disease activity. In fact, the ratio of patients with depression or anxiety in the moderate-to-severe disease activity group is much higher than in the mild disease activity or inactivity group, indicating that lupus disease activity is a risk factor for the severity of depression and anxiety. In line with our study, Nery et al. reported that SLE disease activity measured by SLEDAI was associated with depression severity [18], and Tay et al.[19] and Mak et al. [20] both found that increased SLE activity can be a prediction of more severe anxiety even after adjusting for depressive symptoms. However, Parperis et al. reported that despite the higher SELENA-SLEDAI score in the major depression compared with the group without major depression, this observation was not statistically significant [14] . In contrast, Jarpa et al. conducted a study showing that common mental disorders including depression and anxiety were not associated with lupus disease activity evaluated by SLEDAI [21] . This discordance in studies surrounding the effect of disease activity on depression and anxiety may have been attributed to differences in study methodologies, the diversity in screening instruments employed by the different studies, as well as varying definitions used for depression and anxiety disorders [22] .

The most contributive discovery of our study is the use of the SLEDAI score to predict the likehood of depression and anxiety in patient with SLE. To the best of our knowledge, this is the first study to predict depression and anxiety in SLE patients by disease activity. The discovery of the relationship between depression, anxiety, and SLEDAI score definitely helped rheumatologists better and earlier recognize patients with high risk for mental disorders. In fact, we may estimate depression and anxiety with screening tools in patients with active SLE. And we recommend that screening of depression and anxiety could be a conventional process in SLE patients with SLEDAI scores greater than 8.5. If the GAD-7/PHQ-9 score indicates moderate-severe anxiety/depression, we probably should initiate treatment of anxiety/depression in addition to corticosteroids and immune-suppressive drugs.

Our study had several limitations. First of all, it lacked longitudinal observations of subjects included in this study. Secondly, potential selection bias in who participated in the survey. Thirdly, the correlation of depression/anxiety symptoms with major depression/anxiety was not conducted in this study. Despite the methodological limitations and mixed results presented by the aforementioned studies, our research provides a rationale for future investigations.

## Conclusion

In summary, our study indicates that depression and anxiety are common in Chinese SLE patients. Disease activity is positively related to the severity of depression and anxiety. Those patients whose SLEDAI scores are greater than 8.5 are more likely to suffer from mental disorders which may need conventional depression/anxiety screening and corresponding treatment.

## Data Availability

The datasets used and analyzed during the current study are available from the corresponding author on reasonable request.
